# Valuation of transfusion-free living in MDS: results of health utility interviews with patients

**DOI:** 10.1186/1477-7525-7-81

**Published:** 2009-09-08

**Authors:** Agota Szende, Caroline Schaefer, Thomas F Goss, Kathy Heptinstall, Robert Knight, Michael Lübbert, Barbara Deschler, Pierre Fenaux, Ghulam J Mufti, Sally Killick, Alan F List

**Affiliations:** 1 Covance, Leeds, UK; 2 Covance, Gaithersburg, MD, USA; 3 MDS Foundation, Crosswicks, NJ, USA; 4 Celgene Corporation, Summit, NJ, USA; 5 University of Freiburg, Freiburg, Germany; 6 Hôpital Avicenne, University of Paris XIII, Bobigny, France; 7 King's College Hospital, London, UK; 8 Royal Bournemouth Hospital Foundation Trust, Bournemouth, UK; 9 University of South Florida, Tampa, FL, USA

## Abstract

**Background:**

This study measured how myelodysplastic syndrome (MDS) patients value transfusion independence (TI), reduced transfusions (RT) and transfusion-dependence (TD) using health utility assessment methodology.

**Methods:**

47 MDS patients were interviewed, US (n = 8), France (n = 9), Germany (n = 9) and the UK (n = 21), to elicit the utility value of TI, RT and TD. Health states were developed based on literature; patient forum discussions; and were validated by a hematologist. Face-to-face interviews used the feeling thermometer Visual Analogue Scale (VAS) and the Time Trade-Off (TTO) method to value the health states on a 0 (dead) to 1 (perfect health) scale. Socio-demographic, clinical, and quality-of-life (EQ-5D) characteristics were surveyed to describe the patient sample.

**Results and Discussion:**

The mean age was 67 years (range: 29-83); 45% male, 70% retired; 40% had secondary/high school education, or higher (32%), and 79% lived with family, a partner or spouse, or friends. The mean time from MDS diagnosis was 5 years (range:1-23). Most patients (87%) received previous transfusions and 49% had received a transfusion in the last 3 months. Mean EQ-5D index score was 0.78; patients reported at least some problem with mobility (45%), usual activities (40%), pain/discomfort (47%), and anxiety/depression (34%). Few patients had difficulty understanding the VAS (n = 3) and TTO (n = 4) exercises. Utility scores for TI were higher than for RT (0.84 vs. 0.77; p < 0.001) or TD (0.84 vs. 0.60; p < 0.001). Three patients rated TD worse than dead. Corresponding VAS scale scores were 78 vs. 56; (p < 0.001), and 78 vs. 31 (p < 0.001), respectively.

**Conclusion:**

Patients value TI, suggesting an important role for new treatments aiming to achieve greater TI in MDS. These results can be used in preference-based health economic evaluation of new MDS treatments, such as in future cost-utility studies.

## Background

Myelodysplastic syndromes (MDS) is a term used to describe a group of diseases characterized by ineffective hematopoiesis leading to blood cytopenias and hypercellular bone marrow. MDS has traditionally been considered to be synonymous with 'preleukemia' because of the increased risk of transformation into acute myelogenous leukemia (AML)[1]. Debilitating symptoms of MDS include fatigue, pallor, infection, and bleeding; with commonly associated laboratory findings of anemia, neutropenia, and thrombocytopenia [2,3]. Due to its low incidence and nature, MDS is recognized as an orphan disease by regulatory authorities in Europe and the US.

The majority of MDS patients is unable to maintain normal levels of hemoglobin and is anemic. Consequently, a large proportion of patients rely on frequent red blood cell (RBC) transfusions. While there is currently no widely accepted definition of "transfusion-dependence" with respect to the number and frequency of units received, estimates of the proportion of MDS patients who are transfusion dependent can be up to 80 percent, depending on the type of MDS and disease severity [4]. The International Working Group (IWG) originally standardized response criteria for MDS classifies achieving transfusion independence as a major hematologic improvement and achieving a 50% reduction in transfusions as a minor erythroid response[5].

As new treatments have become available, such as azacitadine for patients with intermediate 2 or high risk and lenalidomide for transfusion-dependent anemia due to Low- or Intermediate-1-risk MDS associated with a deletion 5q cytogenetic abnormality, transfusion independence has become a key treatment objective in everyday clinical practice. Transfusion independence has been associated with a positive impact on health-related quality of life (HRQOL). [6,7] Transfusion independence was also shown to increase the likelihood of survival in a recent retrospective analysis of MDS patients [8].

However, research is lacking on the valuation of health states associated with transfusion independence as opposed to transfusion dependence in MDS patients. The objective of this study was to elicit how MDS patients value transfusion independent living compared to transfusion dependence using validated health utility assessment methods.

## Methods

We performed health utility interviews [9] with a total of 47 MDS patients in France, Germany, the United Kingdom (UK), and the United States (US) to elicit the value of transfusion independence or reduced transfusion burden compared to transfusion dependence (i.e., three distinct health states).

The interviews were performed at one site in each country, except the UK where there were two sites. In Europe, the site selection was facilitated by the MDS Foundation through several of its participating clinical centers that specialize in treating MDS (Paris, Freiburg, London, Bournemouth). In the US, patients were recruited in the Washington, DC area by the Aplastic Anemia MDS Group. Patients had to be currently diagnosed with MDS and be able to read and communicate in the local language. Prior to participating in the research, patients were provided with an information sheet explaining the purpose of the research, and asked to provide written informed consent. Interviews were conducted during the last quarter of 2005 and during 2006.

Health state descriptions were developed based on literature and reports from MDS patient forum discussions, and were validated by a leading clinical expert in the diagnosis and treatment of MDS. A qualitative summary was produced including key concerns that patients commonly brought up in relation to limitations they experienced in various aspects of their HRQOL.

Each health state card included different levels of severity/intensity of problems on the following specific aspects of main HRQOL domains: reliance on blood transfusions and health care provider facility; need to arrange one's life around medical appointments; fatigue and tiredness that limits performance of routine physical activities; interference of disease with social and family life; worry about the future due to health condition; discomfort associated with medical conditions and treatment, and the feeling of being at risk of infection; reliance on support persons for self care and routine activities; feelings of being a burden to family; and feeling sad, hopeless, and helpless.

Table 1 includes the description of the transfusion independent state and transfusion dependent state.

**Table 1:** Health State Descriptions

Transfusion-independent state	You rely on regular medications and routine medical checkups but you do not need to go to a health care facility to receive blood transfusions. You **rarely **feel that you need to arrange your life around medical appointments. You **rarely **experience fatigue and tiredness that would limit you in performing routine physical activities. Your disease **rarely **interferes with your social functioning and family life. You **occasionally **have concerns about your future due to your health. You **periodically **experience **mild to moderate **discomfort associated with health conditions and their treatment, but you rarely feel that you are at risk of infections. You can take care of yourself and routine activities **most of the time**. You **rarely **feel that you are a burden to your family due to your health condition. You **often **feel positive, motivated, and in control of your life despite your health condition.
Transfusion dependent state	You rely on regular blood transfusions and need to spend **significant **time at a health care provider facility. You depend on availability and accessibility of health care facilities and your health care providers. You **often **feel that you need to arrange your life around medical appointments. You **often **experience fatigue and tiredness that limits you in performing routine physical activities. Your disease **often **interferes with your social functioning and family life. You **often **worry about your future due to your health. You experience **moderate to severe **discomfort associated with health conditions and their treatment, and feel that you are at risk of infections. You rely on family or other caregiver support as you **frequently **may need assistance to take care of yourself and routine activities. You may **often **feel that you are a burden to your family due to your health condition. You **often **feel sad, hopeless, and helpless because of your health condition.

The feeling thermometer Visual Analogue Scale (VAS) and the Time Trade-off (TTO) methods were used in face-to-face interviews to value the health states on a scale anchored on 1 (perfect health) and 0 (dead) [10-12]. In the first six patients, the Standard Gamble (SG) method also was administered on a pilot basis together with the TTO, but was then discontinued due to the high rate of patients who did not comprehend the exercise in this predominantly elderly patient population.

In this study, the main aim of the VAS exercise was to help respondents to familiarize themselves with the health states at the beginning of the interview. However, VAS results were also reported separately. The visual prop used was a vertical thermometer-shaped scale, 55 cm long, and numerically scaled in units from 0 to 100. The health states to be rated were printed on cards. The patients were asked to assume that all other aspects of health were normal. They were then asked to place the health states at any point on the scale to correspond to their preferences for these health states.

The TTO part of the interview used a specifically-designed board for the valuation of each MDS condition with varying levels of transfusion dependence. Patients were asked to make a set of paired comparisons between living in the MDS health state for five years, or in perfect health for a shorter period of time. We opted to use five years in the exercise, instead of the most typically used ten-year period, to make the exercise more realistic, given the age and life expectancy of MDS patients. Periods of time in perfect health were varied by 0.5 years. At the point of indifference, where the respondent was unable to choose between 5 years in the MDS health state and the period of perfect health on offer, a utility score was assigned to the MDS state by estimating the ratio of the time in perfect health on offer to the time in the MDS health state. In cases when the respondent had a clear preference between cards but was not willing to sacrifice an additional 0.5 units of time off perfect health, a midpoint score was assigned.

Within each exercise, patients first rated transfusion independence and reduced transfusion health states on the 'perfect health' and worst MDS state (that is, transfusion dependence state) scale, and then rated the transfusion dependence state on the 'Perfect Health' and 'Dead' scale.

The 'chain utility assessment' method was then used to calculate utility scores for each MDS health state (i.e., first anchoring scores on the 'perfect health' and worst MDS scale, and then re-scaling scores on the perfect health and 'dead' scale). As such, the resulted utility scores were anchored on 1 (perfect health) and 0 (dead). The Wilcoxon signed rank test was used to compare TTO utility scores between MDS health states. We also described the distribution of utility scores in order to inform probabilistic analyses to be performed around utilities in any future cost-utility models.

We also administered background questionnaires on socio-demographic, clinical, and HRQOL (using the EuroQOL EQ-5D questionnaire) [13] characteristics to describe the patient sample.

## Results

Key patient demographic data are summarized in Table 2. The majority of patients were retired (70%), had secondary/high school education (40%) or higher (32%), and were living with family, a partner or spouse, or friends (79%).

**Table 2:** Patient Demographic Characteristics

**Age (range)**	67 (29-83) years
**Gender**	45% male

**Country of origin**	

**US**	17% (n = 8)

**France**	19% (n = 9)

**Germany**	19% (n = 9)

UK	45% (n = 21)

The mean time from MDS diagnosis was 5 years (range: 1-23). The majority of patients received blood transfusion(s) previously (87%), and 49% had received a blood transfusion in the last three months.

The mean EQ-5D index value was 0.78 for the patients in our sample, and patients reported at least some problem with mobility (45%), usual activities (40%), pain/discomfort (47%), and anxiety/depression (34%). One patient reported problems with self-care. Patients valued their own health as 0.86 with the TTO method and 0.62 with the VAS method on the 'Perfect Health' and 'Dead' scale.

In the pilot sample of 6 patients, 3 patients did not comprehend the SG exercise and hence this method was discontinued in the rest of the study. In the overall sample, few patients had difficulty understanding the VAS (n = 3) and TTO (n = 4) exercise.

Among valid responses, the transfusion-independent health state was preferred over health states with transfusion requirements using both the VAS and TTO methods (Table 3).

**Table 3:** Ratings for MDS Health States, All Countries

**Health State**	**Method**	
	
	**VAS Method Mean (SD)**	**TTO Method Mean (SD)**
Living in transfusion independence	78 (15)	0.84 (0.16)

Living with reduced transfusion burden	56 (16)	0.77 (0.21)

Living with transfusion dependency	31 (18)	0.60 (0.28)

*P-values for testing the difference between each pair of health states were lower than 0.001.

Paired T-tests showed that VAS scores were statistically significantly higher for the transfusion independent state compared to health states with reduced transfusion requirements (78 vs. 56; p < 0.001) and transfusion dependence (78 vs. 31; p < 0.001). The Wilcoxon signed rank test showed that corresponding TTO scores were 0.84 vs. 0.77 (p < 0.001), and 0.84 vs. 0.60 (p < 0.001), respectively. Using the TTO method, three patients valued transfusion dependence as worse than being dead.

Similar results were observed across countries (Table 4). Differences in utility scores between the transfusion independent and transfusion dependent health states were statistically significant in each country (p < 0.05). However, the difference between health states of transfusion independence and reduced transfusion requirement only reached statistical significance in the UK sample (p = 0.005).

**Table 4:** Ratings for MDS Health States by Country

**Health State**	**Method**	
	
	**VAS Method Mean (SD)**	**TTO Method Mean (SD)**
**France**		

Living in transfusion independence	72 (16)	0.80 (0.16)

Living with reduced transfusion burden	66 (12)	0.70 (0.22)

Living with transfusion dependency	44 (18)	0.56 (0.34)

**Germany**		

Living in transfusion independence	78 (10)	0.80 (0.23)

Living with reduced transfusion burden	52 (13)	0.75 (0.21)

Living with transfusion dependency	23 (18)	0.50 (0.27)

**UK**		

Living in transfusion independence	76 (16)	0.85 (0.15)

Living with reduced transfusion burden	51 (16)	0.77 (0.24)

Living with transfusion dependency	30 (16)	0.65 (0.29)

**US**		

Living in transfusion independence	90 (7)	0.91 (0.07)

Living with reduced transfusion burden	58 (19)	0.84 (0.10)

Living with transfusion dependency	26 (19)	0.61 (0.20)

*P-values for testing the difference between each pair of health states were lower than 0.05. Exceptions were found for the comparison between the transfusion independent and the reduced transfusion health states in the US sample (p = 0.07), the French sample (p = 0.07), and the German sample (p = 0.11).

When excluding responses where patients evaluated all three health states the same (n = 9), TTO-based utility scores for the transfusion independence, reduced transfusion, and transfusion dependence health states in the overall sample were 0.82, 0.73, and 0.52, respectively (p < 0.001).

**Figure 1:**
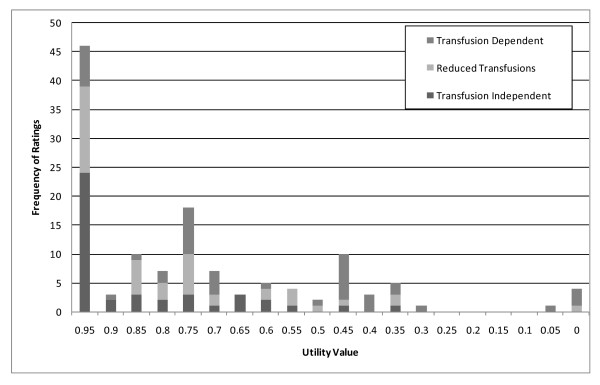
**Distributional Characteristics of TTO Ratings for MDS Health States**.

The frequency of TTO-based utility scores assigned to the three MDS health states are shown in Figure 1. The most frequent utility scores were 0.95 for both the transfusion independence (n = 24) and the reduced transfusion (n = 15) health states. These were responses that reflected a clear preference for perfect health over the MDS health state but responders were not willing to accept a 0.5 year loss in length of life to achieve perfect health, and hence a midpoint of 0.95 utility score was assigned. For the transfusion dependence health state, the most frequently reported utility scores were 0.75 (n = 8) and 0.45 (n = 8).

## Discussion

This study fills in a gap in research by eliciting health utility scores for MDS health states for the first time. Below we discuss the interpretation of actual results, patient sample issues, and methodological considerations.

### Interpretation of results

Previous HRQOL studies have showed that patients with MDS experience impairment in functioning and activities of daily living that result in worse HRQOL than those of similarly aged adults from the general population [14-16]. It was also shown that fatigue caused by chronic anemia has a large impact on the overall HRQOL of MDS patients [7,15]. Transfusion dependency and HRQOL also were shown to be closely associated in MDS patients. Specifically, one cross-sectional study examined the association between transfusion needs and HRQOL, using the disease-specific QOL-E questionnaire in 52 MDS patients [6]. The study showed that even after partially controlling for hemoglobin level and other clinical variables, the number of transfusions per month was inversely correlated with HRQOL. Patients who did not receive monthly transfusions because their hemoglobin level was higher than 8 g/dL had significantly higher overall HRQOL scores than transfusion-dependent patients.

Our study results provide important new evidence that independence from transfusions is not only associated with better HRQOL scores but patients also put a high value on being transfusion-free when their preferences are measured on a utility scale. This finding is significant as it indicates that transfusion dependency is regarded as 'bad enough' by MDS patients to be willing to trade-off length of life in order to achieve transfusion independence. The sacrifices that patients were willing to make were the most substantial for the transfusion dependence state reflected by the 0.60 utility score.

While the our results for each pair of health states in the overall sample and for the transfusion independent versus transfusion dependent health states in each individual country were statistically highly significant, the observed difference between the transfusion independent and reduced transfusion requirement health states was only statistically significant in the UK country sample. For this reason, the use of country specific MDS utility scores should be carefully considered and analyst may decide to use the pooled results in base case or sensitivity analysis in economic evaluations in countries where differences did not reach statistical difference between each pair of health states.

Although there is a lack of utility studies in MDS with which to compare our results, comparison of MDS health states with utility scores for the general population can be made with some limitations. Comparability is limited by the fact that patients tend to report better scores for disease states than would a person from the general population without the condition. In addition, available utility norms for general populations were elicited with the TTO-based generic utility questionnaire, the EQ-5D, instead of using direct TTO assessments like our study did. Nevertheless, average health utility scores for the general population were reported as 0.86 and 0.87 for the UK and the US, respectively [17,18]. Corresponding utility scores for people between the age of 65-74 were 0.78 and 0.82, respectively. The comparison between these utility scores and those for MDS health states from our study confirms that transfusion-dependent MDS is valued as a condition with substantially reduced health status.

### Patient sample issues

Studies involving MDS patients, especially those on health outcomes, have traditionally captured small samples of patients due to the rareness of the condition and the lack of new treatments under evaluation. MDS patients are typically treated in centers that specialize in MDS. We tried to achieve a reasonably representative sample of MDS patients from a selection of these centers that also represent cultural differences across four countries and demographic characteristics, such as age and gender.

Despite these efforts and achieving results that are statistically significant, some limitations need to be noted. The US sample was recruited from a patient organization. As such, these patients may have not been fully representative of the overall patient population. Members of patient organizations may be milder in disease severity and may represent different views than non-members. Data from the administration of the background HRQOL questionnaire, the EQ-5D, suggested that MDS patients in our survey may had currently a milder MDS disease severity as their EQ-5D scores were only somewhat worse than that of the general population. While most patients came from the elderly age groups typical in MDS, there were two patients in the UK who were unusually young for MDS (32 and 33 years old). Our sample also seemed to include a lower transfusion-dependency rate than observed in many MDS sub-types with 49% of patients receiving a transfusion in the past 3 months. However, 87% of patients in our sample had received blood transfusion before, suggesting that they were able to give informed responses when rating the transfusion dependency health state.

The administration of the background HRQOL questionnaire also enabled us to compare our patient sample with that of the general population. As expected, our patient sample presented worse HRQOL ratings than the general population. For example, the proportion of people reporting at least some problem in a comparable age-group from a UK-based general population sample was lower than observed in our sample, including rates of reported problems of 29% (mobility), 25% (usual activity), 46% (pain/discomfort) and 28% (anxiety/depression) [19].

### Methodology considerations

To elicit health utility values associated with health states, a number of different methods exist. These methods can differ in key aspects such as the preference elicitation technique used or the sample whose values are measured [20]. The main utility results that our study reported were based on the TTO method, which is one of the two most widely accepted choice-based valuation techniques [12]. In interpreting results, one needs to bear in mind that utility scores using other methods may have given different scores. Specifically, as noted in the literature, the SG technique typically yields higher, while the VAS technique typically yields lower, ratings than does the TTO technique [21]. Our results on lower VAS scores than TTO scores for MDS health states were consistent with this general finding. Patients' valuation of their own current health also yielded consistently higher values with the TTO method compared to VAS.

We note that variations within utility elicitation methods also exist regarding the exclusion criteria used for valid responses or the valuation of health states worse than 'dead'. Specifically, the base-case results of our study included responses that gave the same TTO ratings for each of the three health states, as long as the respondent comprehended the exercise. However, we also reported that utility scores for each health state were lower when these responses were excluded. Utility scores that we reported for this scenario may be of interest to those who believe that the TTO method should be able to differentiate preferences for different health states in each respondent. Health states regarded worse than dead were assigned a utility score of zero in our study. Should a method that assigns negative scores to these valuations have been applied, scores for the RT and in particular for the TD state would have been even lower than reported here.

There also is a debate regarding whose values utility scores should reflect. Some researchers argue that valuations by patients are more informed as they have direct knowledge of the condition; while others advocate that views of the general population should be taken into account when utility results are used to inform resource allocation in public health care systems [20]. In practice, utility valuation studies on disease-specific health states are more often done in the respective patient sample, like our study did. However, our results may be interesting to compare with utility scores gained via generic utility instruments that reflect the general public's values and are administered among MDS patients in any future studies of that nature.

## Conclusion

The results of this study show that patients associate a high value with achieving transfusion independence in MDS. These findings suggest an important role for new treatments aimed at achieving greater transfusion independence in MDS. New health utility values elicited in this study could be used in health economic evaluations of emerging MDS treatments that express and compare the health outcome of therapies in quality-adjusted life years (QALYs).

## Competing interests

The authors declare that they have no competing interests.

## Authors' contributions

AS prepared study design, developed MDS health state descriptions, carried out study, analysis, and report preparation. CS carried out study, analysis, and report preparation. TFG developed MDS health state descriptions and carried out the analysis. KH carried out the study. RK contributed to the study design. ML carried out the study. BD carried out the study. PF carried out the study. GJM carried out the study. SK carried out the study. AFL developed MDS health state descriptions and contributed to study design. all authors read and approved the final manuscript.
